# Systemic Therapy for Youth at Clinical High Risk for Psychosis: A Pilot Study

**DOI:** 10.3389/fpsyt.2017.00211

**Published:** 2017-10-20

**Authors:** Jingyu Shi, Lu Wang, Yuhong Yao, Chenyu Zhan, Na Su, Xudong Zhao

**Affiliations:** ^1^East Hospital, School of Medicine, Tongji University, Shanghai, China; ^2^Students Counseling Center, Tongji University, Shanghai, China; ^3^School of Medicine, Tongji University, Shanghai, China

**Keywords:** systemic therapy, intervention, clinical high risk, psychosis, youth

## Abstract

Psychosocial intervention trials for youth at clinical high risk (CHR) for psychosis have shown promising effects on treating psychotic symptoms but have not focused on psychosocial functional outcomes, and those studies have been conducted among help-seeking patients; there is a lack of research on non-clinical young CHR individuals. Systemic therapy (ST) is grounded in systemic-constructivist and psychosocial resilience theories. It has a number of advantages that makes it attractive for use with CHR individuals in non-clinical context. The present study evaluated the effect of ST for students at CHR on reducing symptoms and enhancing psychosocial function. This was a single-blind randomized controlled trial for CHR young people comparing ST to supportive therapy with a 6-month treatment. Psychotic and depressive symptoms (DS) as well as self-esteem and social support (SS) were assessed at pre- and posttreatment. 26 CHR individuals were randomly divided into intervention group (*n* = 13) and control group (*n* = 13). There were no significant differences in severity of symptoms, level of SS and self-esteem at baseline between the two groups (*P* > 0.05). At posttreatment, significant improvements in positive and DS as well as SS and self-esteem were observed in the ST group (*P* < 0.05); in the control group, these improvements were not significant (*P* > 0.05). The findings indicated that systemic intervention for university students at CHR for psychosis may have a positive effect on symptoms and self-esteem as well as SS in short term. More long-term research is needed to further evaluate this intervention.

## Introduction

Young people are at greater risk of developing mental illness as they transition from childhood to adulthood (1). Psychosis typically emerges in late adolescence or early adulthood and can disrupt social and psychological development, including the attainment of educational goals and relationship skills, thus seriously impairing their quality of life. Prior to the first onset of psychotic disorder, 80–90% of individuals experience attenuated psychotic symptoms, and this stage was conceptualized as a prodromal phase, or at clinical high risk (CHR) (2, 3). The CHR for psychosis state is characterized by the presence of low intensity/frequency psychotic symptoms, brief limited psychotic episodes, and/or familial risk and/or schizotypal personality disorder in the presence of psychosocial functional decline, and with increased risk of developing psychosis (4). The CHR criteria provide an important opportunity for early intervention in preventing or delaying the onset of psychosis and reducing the social and economic burden associated with long-term mental health problems.

Systemic therapy (ST) has shown particular promise in improving adolescent and adult mental health problems (5–7). ST is based on system theory and controlling theory. It emphasizes on viewing problems in developing, comprehensive, positive, and diverse ways and focuses on understanding the individual symptoms within the system of interpersonal relationships. In ST, the function and significance of the symptoms is much valued. Attention will be paid to the interaction between individual and his/her environment. System therapy is suitable for individuals and families. Its value orientation is positive psychology, namely, resource orientation, treating the patients/clients as experts of solving their problems; it is assumed that the patients/clients do have the resource for solving the problem. ST emphasizes and explores the individual strengths, ability, ideas, and social resources, focusing on extending the space beyond the problem, to bring new and diverse perspectives for individuals and families, and thus to promote changes from the inner and interpersonal levels (8). Previous research reported that ST had shown positive effects on improving the symptoms and psychosocial functioning of schizophrenia patients (9). In addition, ST considers changes outside of the treatment; all in all, it is an efficient, economical, short-term treatment with a long interval.

To our knowledge, there was no specific manual for the treatment of clinical risk of psychosis available at the time of research, therefore a manual of ST was developed by the experts for ST, integrating a broad range of systemic methods, for the CHR university students.

We reviewed ST manuals for psychotic disorders in adult psychotherapy (10) and well-established ST manuals for various disorders (11–13). We used general ST concepts (8), integrating constructivist, solution-oriented methods (14), in addition to paying attention to the disorder-specific relational systemic dynamics (15, 16). According to the literature and to our experiences of treating patients, the aim of ST is to contextualize attenuated psychotic symptoms by addressing an individual’s social system to which he/she attaches importance. Although the social system members could not attend the meeting physically, circular questions included them on a cognitive level. The analysis of transgenerational relationships and of past and present interpersonal interactions help to develop a new understanding of the important roles, places, and resources of all system members. Under the systemic model, the therapy sessions alike are held individually but with a strong focus on relationship issues. The ST manual differentiates between four phases, which are described in detail in the Section “Materials and Methods.”

We found only seven systemic intervention RCTs for psychotic disorder (17–23), and only one for patients at CHR state (22). This study (*n* = 40, China) compared only medication treatment with 10 sessions of structural family therapy (one school of ST) plus medication. The outcome relied on the family function, severity of prodromal symptoms, and treatment compliance. This trial demonstrated the structural family therapy group with a higher reduction of severity of symptoms, higher treatment compliance, and a higher improvement in family function than the control group (*P* < 0.05).

To our knowledge, there is a lack of studies, which explore the effectiveness of ST for CHR individuals among general populations. Most studies focus on the primary outcome as a dichotomous one of transition to psychosis, rather than the dimensional domains of psychosocial functioning. Previous studies indicate that the adolescents and young adults at CHR might show poor psychosocial functioning. In some CHR studies, it was reported that the subjects had shown a lower level of self-esteem and social support (SS) in comparison to healthy controls (24, 25), and the levels of self-esteem and SS were negatively associated with the severity of the prodromal psychotic symptoms (25–28). However, many studies indicated that such psychosocial factors could play a positive role in mental health (29–31). Moreover, it is considered as an important resource for coping with psychotic symptoms (32). Thus, a positive self-perception and a strong sense of control would prevent a negative perception of daily stress; self-esteem also has a stress-buffering effect, which protects youth from the harmful effects of stress on mental health (33). In psychotic disorders, low self-esteem has been demonstrated in both the development of delusions (34) as well as in the maintenance of psychotic symptoms (35). SS as another important personal resource has been improved, having positive effects on mental health by either directly enhancing self-esteem or indirectly by protecting individuals against the adverse impact of exposure to stress and trauma (36). SS may exert such positive effects both prior to and at the onset, as well as during the course of disorder, operating to reduce both risks of onset and relapse.

Therefore, this study was designed to investigate the effectiveness of ST for CHR individuals among general youth populations on both symptom and psychosocial functioning outcome. We hypothesize that compared to the control group, ST would reduce symptom severity while improving self-esteem as well as SS.

## Materials and Methods

### Study Design

This study was a single-blind RCT of ST compared to supportive therapy conducted at university with a 6-month treatment. The treatment began within 2 weeks of completion of the baseline assessment and was available for up to 10 sessions over a 6-month period. Assessments were conducted after the treatment. Clinical raters were blind to treatment groups.

### Participants

In the study participated 26 university students at CHR (12 males and 14 females; age 18.85 ± 1.120). The 26 CHR subjects were screened out from 2,800 students of the first and second grades. The status of CHR was diagnosed through a two-stage assessment, consisting of screening with the self-report Chinese version of 16-item Prodromal Questionnaire (CPQ-16) (37) and an assessment interview according to the Structured Interview for Psychosis-Risk Syndromes (SIPS). The criteria of prodromal symptoms require that individuals meet at least one of the three clinical criteria: (1) attenuated positive symptom prodromal syndrome, defined as recent occurrence of attenuated positive psychotic symptoms with sufficient frequency or severity; (2) brief intermittent psychosis prodromal syndrome, defined as recent presenting of psychotic symptoms with spontaneous remission within 1 week; and (3) genetic risk and deterioration prodromal syndrome, defined as coexisting of genetic risk and recent functional decline (38). This study had the ethics approval from the institutional review board of Tongji University. Prior to the study, each participant provided written informed consent.

### Procedure

In order to select the potential individuals at CHR for psychosis, at first stage, the CPQ-16 was given to 2,800 university students. In the current study, the positive threshold for the CPQ-16 was set at 6, according to the study conducted by Ising et al. (39). Totally, 611 students reached this cut-off score, and they were invited to take part in our second stage for SIPS. We received agreement from 529 participants who accepted the SIPS, which was operated by trained psychiatrists. Of the 529 students referred to the trial, 32 were screened and 26 were randomized for treatment, 13 to the ST group and 13 to the control group. The 26 students at CHR completed the 6-month treatment.

### Measures

#### Symptoms

The severity of positive and negative symptoms (NS) was measured with the Scale of Prodromal Syndromes from 0 (absent) to 6 (psychotic) and a total symptom score was created (38). The Montgomery–Åsberg Depression Rating Scale was used to assess depressive symptoms (DS) (40). The overall functioning was rated by the Global Assessment of Functioning scale (GAF) (41).

#### Self-Esteem

Self-esteem was rated with the self-reported Rosenberg Self-esteem Scale (SES) (42), which includes 10 items with a measure of global self-esteem. Each item is rated on a 4-point Likert scale ranging from 1 = strongly disagree to 4 = strongly agree. It has been demonstrated that the RSES has good reliability and validity (43, 44).

#### Social Support

Social support was measured with the Perceived Social Support Scale (45), which is based on the unique social and cultural conditions in China. The scale is made up by 12 items designed to assess subjective SS rated on a 7-point Likert scale ranging from 1 = strongly disagree to 7 = strongly agree. The higher scores reflect higher SS. The scale has been widely used among Chinese populations and proved to have a good validity and reliability (44, 45).

### Interventions

Systemic therapy for CHR students followed the manual developed according to Carr (7) by experts on ST, ranging from 10 to 30 years of experience. It is grounded in systemic-constructivist (46) and psychosocial resilience theories (47) and based on a solution focused model that prioritizes a careful clarification of therapeutic goal. It is solution and resource oriented, reframing one’s problem from functional and meaningful perspectives, using a variety of questioning techniques to enrich perspectives toward identified problems, exploring and strengthening the resource of the clients and creating more space and possibilities to solve the problem, homework tasks were usually given to help clients gain new insights and experiences. The treatment comprises four phases, each phase including 2 to 5 sessions. First phase: establishment of therapeutic relationship, collection of information, and clarification of therapeutic goals; second phase: understanding the context of the identified problem as well as interactive patterns around the identified problem, reconstruction of the problem, and exploring resources and solutions, making use of the resources and putting the solution into practice; third phase: reinforcement and deepening of changes; fourth phase: relapse prevention. Each session lasted 50 min. During the process of treatment, the treatment interval gradually extended from weekly to monthly. The details of sessions are listed in Table 1.

**Table 1:** Topics and key concepts of systemic therapy.

Therapeutic phases	Topics	Key issues and techniques	Homework
First phase (2 sessions)	Introduction and join in	Buildup rapport	Write a strength and resource list, including at least 50 points
	Collection of information	Positive listening, systemic questioning, buildup the first hypotheses, draw genogram	Write down 10 things, which the clients want to do most in the next 3 years
	Clarification of therapeutic goals	Inquiry about the expectations, using systemic questioning to clarify the therapeutic goal, which is clear, feasible, and in a positive way of formulation	

Second phase (5 session)	Understanding the context of the identified problem as well as interactive pattern around the identified problem	Shifting the pathology from symptoms to relations. Understanding the meaning and function of the identified problem in an interpersonal system; systemic questioning such as circular questions, exception questions, scaling questions; family boards and timelines	On odd days, the client should act as if the problems become more serious and, on even days, the client should act as if the problems disappear, and meanwhile, he or she observes the reaction of others
	Reconstruction of the problem and exploring resources and solutions	Finding out and creating diverse possibilities; challenging the certainty of the knowledge of the identified problem; rewriting the self-narrative and reframing, positive connotation	
	Making use of the resource and putting the solution into practice	Homework	

Third phase (2 session)	Reinforcement and deepening of changes	Reflecting and reviewing the progress and changes; expand the details of the changes; emphasize the client’s efforts and abilities to make changes; discuss about how to maintain the changes	Observe and write down the sympathetic behaviors

Fourth phase (1 session)	Relapse prevention	Considering the risks of relapse and building up a treasure box of strategies	Build up a treasure box of strategies

Supportive therapy is conducted only using general counseling techniques: warm, empathic, and non-judgmental face-to-face contact and supportive listening.

All therapy sessions including ST and supportive therapy were delivered by a qualified systemic therapist with 10 years experience. It has the advantage of controlling for non-specific aspects of treatment (e.g., therapist age, sex, personality, therapist experience). The therapist received expert and peer supervision regularly.

### Data Analysis

All statistical procedures were conducted using the Statistical Package for the Social Sciences (SPSS 17.0). Chi-square tests were applied to compare categorical demographic variables between the two groups. *T*-tests for independent samples were used to assess differences in self-esteem score and SS score between the two groups. *T*-tests for paired samples as well as effect sizes were calculated to assess differences in self-esteem score, SS score, symptom score, and functioning (GAF) between baseline and posttreatment in two groups. End of intervention scores on various outcome measures between the ST group and the control groups were compared using ANOVA of repeated measures to explore the impact effects of time and intervention.

## Results

### Social Demographic Characteristics and Baseline Data

There were no significant differences in age, gender, whether only child and family history between the two groups (*P* > 0.05). There were no significant differences in severity of psychotic symptoms, overall functioning, level of supports, and self-esteem at baseline between the two groups (*P* > 0.05). Social demographic characteristics and baseline data are presented in Table 2.

**Table 2:** Baseline clinical and social demographic characteristics of two groups.

	ST (*n* = 13)	Control (*n* = 13)	*t/X*^2^	*P*
Age mean (SD)	18.85 (0.987)	18.85 (1.281)	0.000	1.000
Female (%)	9 (64.2)	5 (38.5)	2.476	0.116
Only child (%)	11 (84.6)	10 (76.9)	0.248	0.619
Family history (%)	0 (0.0)	1 (7.7)	1.040	0.308
PS	6.85 (3.460)	7.62 (3.477)	−0.565	0.577
NS	4.54 (4.666)	3.92 (3.499)	0.380	0.707
DS	6.62 (5.455)	7.08 (6.849)	−0.190	0.851
Global assessment of functioning scale	73.62 (5.546)	72.85 (6.453)	0.326	0.747
Self-esteem	26.54 (4.824)	28.08 (4.192)	−0.868	0.394
Social support	56.38 (12.868)	62.46 (8.491)	−1.421	0.168

*ST, systemic therapy; PS, positive symptoms; NS, negative symptoms; DS, depressive symptoms; SE, self-esteem; SS, social support*.

### Effectiveness of Systemic Intervention on Severity of Symptoms and GAF

**Figure 1:**
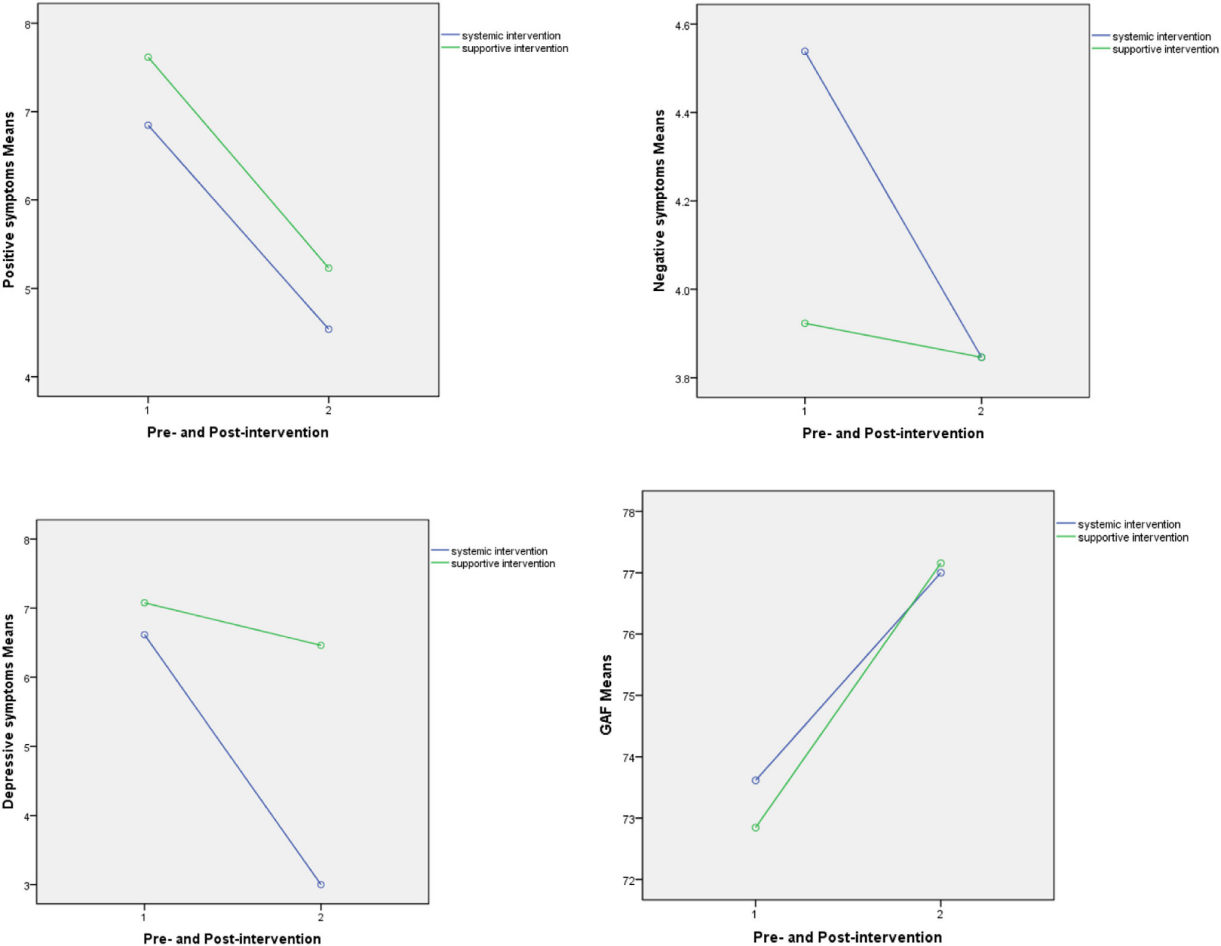
Improvements in scores of severity of symptoms.

Participants in the ST group demonstrated significant decreases in severity of positive symptoms (PS) and DS comparing to that at baseline (*P* = 0.005); however, the participants in the control group had shown no significant changes in severity of PS and DS (*P* > 0.05). Improvements were also observed in NS and GAF in both groups, but the changes were not significant (*P* > 0.05). The results are presented in Table 3. Furthermore, the statistic analysis results of ANOVA of repeated measures didn’t show significant impact effects of the time factor, interaction between time and intervention, as well as intervention factor on the changes. These interactions are graphed in Figure 1. The statistic results are presented in Table 4.

**Table 3:** Comparison of changes between groups.

Measures		Pre-intervention	Post-intervention	Cohen’s *d*	*t*	*P*
Positive symptoms	Systemic therapy (ST)	6.85 (3.46)	4.54 (5.08)	0.53	3.426	0.005
	Control	7.62 (3.48)	5.23 (3.90)	0.65	1.934	0.077
NS	ST	4.54 (4.67)	3.85 (4.78)	0.15	0.454	0.658
	Control	3.92 (3.50)	3.85 (4.67)	0.02	0.054	0.958
Depressive symptoms	ST	6.62 (5.46)	3.00 (4.08)	0.75	3.065	0.010
	Control	7.08 (6.85)	6.46 (8.24)	0.08	0.211	0.837
Global Assessment of Functioning scale	ST	73.62 (5.55)	77.00 (9.57)	0.43	−1.206	0.251
	Control	72.85 (6.45)	77.15 (10.01)	0.51	−1.510	0.157
SE	ST	26.54 (4.82)	29.08 (3.73)	0.59	−2.980	0.011
	Control	28.08 (4.19)	28.23 (5.09)	0.03	−0.148	0.885
SS	ST	56.31 (12.76)	62.54 (14. 81)	0.45	−2.916	0.013
	Control	62.46 (8.49)	62.08 (12.47)	0.04	−0.464	0.901

**Table 4:** ANOVA of repeated measures.

Measures	Difference between pre- and post (time)		Time × group		Difference between groups	

	*F*	*P*	*F*	*P*	*F*	*P*
Positive symptoms	11.157	0.003	0.003	0.957	0.266	0.611
NS	0.137	0.715	0.087	0.770	0.049	0.827
Depressive symptoms	1.806	0.192	0.908	0.350	1.033	0.320
Global Assessment of Functioning scale	3.695	0.067	0.053	0.820	0.015	0.902
SE	4.025	0.056	3.158	0.088	0.045	0.833
SS	4.797	0.038	2.154	0.155	0.665	0.423

### Effectiveness of ST on Level of Self-Esteem and SS

**Figure 2:**
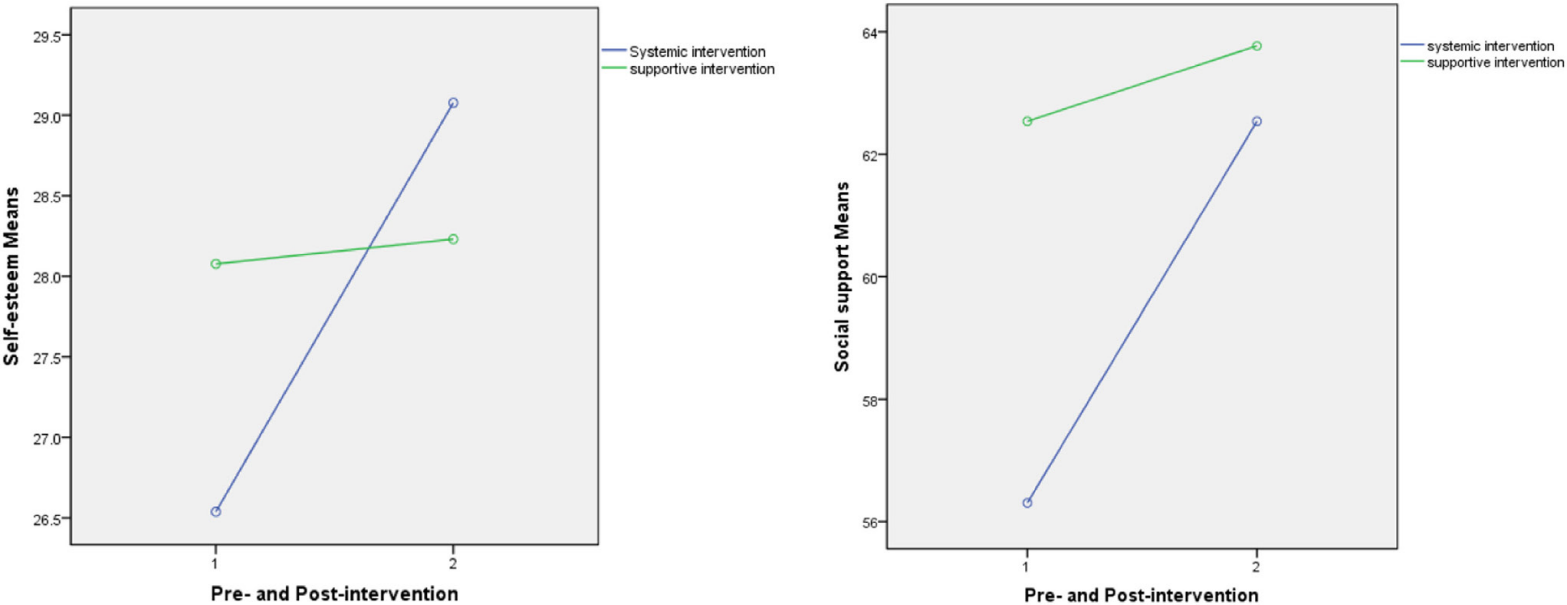
Improvements in level of self-esteem and social support.

Obvious improvements in level of SS and self-esteem in participants in ST were evident at posttreatment (*P* = 0.013, *P* = 0.011); the level of SS as well as self-esteem of the individuals in the control group had no significant changes at posttreatment (*P* > 0.05). The results are presented in Table 3. Furthermore, the statistic analysis results of ANOVA of repeated measures didn’t show significant impact effects of the time factor, interaction between time and intervention, as well as intervention factor on the changes. These interactions are graphed in Figure 2. The statistic results are presented in Table 4.

### Clinical Significance

At posttreatment, attenuated psychotic symptoms in eight participants (61.5%) in the ST group and six participants (46.2%) in the control group were reduced to a level of remission from an initial CHR status as defined by the SIPS. However, the difference was not significant (*X*^2^ = 0.619, *P* = 0.431). One CHR individual in the control group developed an onset of the psychotic manic episode of bipolar I disorder during the follow-up period. The conversion rate was 3.8% (1/26).

## Discussion

The goals of this study were (1) to evaluate the effectiveness of systemic family therapy focusing specifically on the change in youth-attenuated psychotic symptoms and improvements in psychosocial functioning, and (2) to evaluate the effectiveness of systemic family therapy for CHR individuals in a non-clinical context. Most previous systemic intervention studies evaluated its impact on symptoms and treatment compliance. Although in clinical context to reduce the symptoms and enhance the treatment compliance has a significant benefit for youth and families, it is important to know if ST enhances the psychosocial functioning level and the feasibility in non-clinical context. These issues provided the impetus for the present study. As far as we know, this trial is the first RCTs of ST for young people at CHR for psychosis. The trial targeted a non-clinical young sample, evaluated the treatment effect of ST, and employed an active control condition.

As expected, we found that ST led to a significant reduction in positive and DS and to an obvious improvement in self-esteem and SS comparing between the pre-and posttreatment measure outcomes. The ecological model, which underpins ST proposes that individuals’ problems become problems only in the context and different social systems. The uniqueness of ST lies in its innovative nature of reconstructing “problems” in developing, comprehensive, positive, and diverse ways, treating the patients/clients respectfully as experts of solving their problem, and suggesting creative, unusual homework tasks, which function as perturbacion in behavioral and interactive patterns. These systemic therapeutic concepts are quite suitable for the non-clinical context. First, ST focuses on resources rather than on deficits, and it recognizes the expert status of the young people at CHR. This is in line with the view of resilience in CHR individuals that focuses on protective factors against adversity (48). Second, in a systemic constructivist perspective, psychotic symptoms are regarded as phenomena, which are neither from the beginning as surely existent confirmed nor as definitively non-existent considered. Symptoms could be considered as psychological and biological phenomena, but also as deriving from social interaction constructed reality. Therefore, this understanding about the symptoms comes as a great relief to the students at CHR and suggests that they could influence their symptoms. Third, the approach moves the focus from problems to solutions by setting limited and clearly defined goals, and it promotes an early and positive relationship between students and therapist. ST can give them support in resolving or at least reducing such problems through a structured and focused way, emphasizing the individual’s unique contribution. In systemic intervention, the students at CHR could gradually develop a resource-oriented mindset: focusing on resources rather than deficits, on solutions rather than problems, and that could contribute to enhancing the SS and self-esteem, and thus, they are playing a positive role in improvement of the symptoms.

From Table 4, it could be seen that there are no statistically significant differences when considering the interaction between the time and group. There are several potential explanations for it. First, there may have been a “floor effect” with little room for self-reported, treatment-related improvement, because youth did not perceive themselves to have clinically significant problems. Second, because of the small sample size, the quantitative study design and statistical analyses could not fully reflect the difference in quality of changes between groups. Third, the natural recovery process is an essential factor to influence the clinical outcomes. In some cases, personal qualities, such as resilience might play a more important role to protect a subject’s mental health (49).

All in all, we can say that the results of this pilot study support at least the possibility of using a systemic intervention for young people at CHR in non-clinical context. Nevertheless, it is necessary to do further research in this area with larger sample sizes, standardized measures, prolonged follow-up assessments, and an exploration of effective therapeutic factors on improvement of self-esteem as well as SS. Specifically, we suggest to investigate how self-esteem and SS reduce symptoms or increase positive reactions and to compare this with other established therapies.

## Ethics Statement

Our study had ethics approval from the School of Medicine Ethics Committee, Tongji University, Shanghai, China. Reference number: 2014YXY16, and we acquired written informed consent from participants of the study.

## Author Contributions

JS contributed to study design, recruitment of participants, data analysis and interpretation, and writing of the manuscript. LW contributed to recruitment of participants and interpretation of results. YY, CZ, and NS contributed to recruitment of participants. XZ contributed to study design and interpretation of results.

## Conflict of Interest Statement

The authors declare that the research was conducted in the absence of any commercial or financial relationships that could be construed as a potential conflict of interest.
